# Metagenomic Sequencing of Microbial Communities from Brackish Water of Pangong Lake of the Northwest Indian Himalayas

**DOI:** 10.1128/genomeA.01029-17

**Published:** 2017-10-05

**Authors:** Rashmi Rathour, Juhi Gupta, Madan Kumar, Moonmoon Hiloidhari, Anil Kumar Mehrotra, Indu Shekhar Thakur

**Affiliations:** a School of Environmental Sciences, Jawaharlal Nehru University, New Delhi, India; b Chemical and Petroleum Engineering, Schulich School of Engineering, University of Calgary, Calgary, Alberta, Canada

## Abstract

Pangong is a brackish water lake having environmental conditions that are hostile to supporting life. This is the first report unveiling the microbial diversity of sediment from Pangong Lake, Ladakh, India, using a high-throughput metagenomic approach. Metagenomic data analysis revealed a community structure of microbes in which functional genetic diversity facilitates their survival.

## GENOME ANNOUNCEMENT

Sediments are rich sources of microbial diversity and represent a special realm in aquatic environments (1). To overcome the limitations of the culture approach in studying these organisms, culture-independent approaches like metagenomics are applied to characterize microbial communities, discover novel genes, and analyze metabolic pathways directly from the environment (2, 3). There is very limited information available on the microbial diversity present at high-altitude cold habitats of the Himalayas (4). The present study investigates, through a metagenomic approach, the functional genetic diversity of microbes present in Pangong Lake, a large brackish water lake situated at a height of 4,250 m above mean sea level in the Himalayas. The microbes present there are halotolerant and cold adapted, and identifying the diversity of the novel cold-active enzymes and secondary metabolites assisting in the survival of these microbes may have great biotechnological potential.

The sediment samples were obtained from Pangong Lake (33°43′04.59ʺN:78°53ʹ48.48ʺE), Ladakh, J&K (Jammu and Kashmir) India, in September 2016 and stored at 4°C until further analysis. The DNA was extracted using the Exgene soil DNA kit (GeneAll Biotechnology Co., Ltd.), and sequencing was performed on the Illumina platform. The paired-end sequencing libraries (2 × 150 bp) were prepared using the Illumina TruSeq Nano DNA library prep kit and were sequenced on the Illumina NextSeq500 platform. The raw data were processed to obtain high-quality clean reads (quality value >20) using Trimmomatic version 0.35 (5). The filtered high-quality reads of the sample were assembled into scaffolds using CLC Genomics Workbench, and genes were predicted using Prodigal version 2.6.3 with default parameters (6). Taxonomic analysis of the predicted genes was carried out using Kaiju (7), a program for sensitive taxonomic classification of high-throughput metagenomics sequencing data. Cognizer (8), which is a comprehensive stand-alone framework that simultaneously provides COG (9), KEGG (10), Pfam (11), GO (12), and SEED (13) subsystem annotations to individual sequences constituting metagenomics data sets, was used for performing the functional analysis of the genes.

The mean of the library fragment size distribution was 486 bp, and ∼3 Gb of high-quality data were obtained, with 10,386,213 reads assembled into scaffolds. After assembly, the total size of the scaffolds was 248,068 bp, with an *N*_50_ value of 635 bp, and 337,527 genes, with an average gene length of 401 bp, were predicted. The predicted genes having a length of <300 bp were discarded from taxonomical analysis and functional classification. Taxonomical classification was as follows: bacteria (83.86%), archaea (0.24%), eukaryotes (0.42%), viruses (0.41%), and unclassified (15.02%). The major phyla represented were *Proteobacteria* (54.36%), *Bacteroidetes* (24.01%), *Firmicutes* (1.14%), *Actinobacteria* (0.85%), *Balneolaeota* (0.79%), *Cyanobacteria* (0.59%), *Verrucomicrobia* (0.47%), *Euryarchaeota* (0.21%), *Planctomycetes* (0.19%), and *Ascomycota* (0.10%). At the genus level, *Methylophaga* (10.19%) was found to be the most abundant. Functional analysis of the sequence classified most of the data as being related to carbohydrate metabolism, energy metabolism, lipid metabolism, and nucleotide metabolism.

Metagenomic analysis revealed a diverse domain of microbial communities thriving in harsh conditions, creating a base for further microbial exploration to improve the efficacy of bioprospecting metagenomics of soil and sediment, which may lead to the discovery of novel enzymes and bioactivities.

### Accession number(s).

The nucleotide sequences reported here have been submitted to the NCBI Sequence Read Archive (SRA) under accession number SRX2861366.
